# Artificial Intelligence–Enabled Social Media Analysis for Pharmacovigilance of COVID-19 Vaccinations in the United Kingdom: Observational Study

**DOI:** 10.2196/32543

**Published:** 2022-05-27

**Authors:** Zain Hussain, Zakariya Sheikh, Ahsen Tahir, Kia Dashtipour, Mandar Gogate, Aziz Sheikh, Amir Hussain

**Affiliations:** 1 Edinburgh Medical School College of Medicine and Veterinary Medicine University of Edinburgh Edinburgh United Kingdom; 2 School of Medicine University of Dundee Dundee United Kingdom; 3 Department of Electrical Engineering University of Engineering and Technology Lahore Pakistan; 4 School of Computing Edinburgh Napier University Edinburgh United Kingdom; 5 Usher Institute College of Medicine and Veterinary Medicine University of Edinburgh Edinburgh United Kingdom

**Keywords:** COVID-19, artificial intelligence, deep learning, Facebook, health informatics, natural language processing, public health, sentiment analysis, social media, Twitter, infodemiology, vaccination

## Abstract

**Background:**

The rollout of vaccines for COVID-19 in the United Kingdom started in December 2020. Uptake has been high, and there has been a subsequent reduction in infections, hospitalizations, and deaths among vaccinated individuals. However, vaccine hesitancy remains a concern, in particular relating to adverse effects following immunization (AEFIs). Social media analysis has the potential to inform policy makers about AEFIs being discussed by the public as well as public attitudes toward the national immunization campaign.

**Objective:**

We sought to assess the frequency and nature of AEFI-related mentions on social media in the United Kingdom and to provide insights on public sentiments toward COVID-19 vaccines.

**Methods:**

We extracted and analyzed over 121,406 relevant Twitter and Facebook posts, from December 8, 2020, to April 30, 2021. These were thematically filtered using a 2-step approach, initially using COVID-19–related keywords and then using vaccine- and manufacturer-related keywords. We identified AEFI-related keywords and modeled their word frequency to monitor their trends over 2-week periods. We also adapted and utilized our recently developed hybrid ensemble model, which combines state-of-the-art lexicon rule–based and deep learning–based approaches, to analyze sentiment trends relating to the main vaccines available in the United Kingdom.

**Results:**

Our COVID-19 AEFI search strategy identified 46,762 unique Facebook posts by 14,346 users and 74,644 tweets (excluding retweets) by 36,446 users over the 4-month period. We identified an increasing trend in the number of mentions for each AEFI on social media over the study period. The most frequent AEFI mentions were found to be symptoms related to appetite (n=79,132, 14%), allergy (n=53,924, 9%), injection site (n=56,152, 10%), and clots (n=43,907, 8%). We also found some rarely reported AEFIs such as Bell palsy (n=11,909, 2%) and Guillain-Barre syndrome (n=9576, 2%) being discussed as frequently as more well-known side effects like headache (n=10,641, 2%), fever (n=12,707, 2%), and diarrhea (n=16,559, 3%). Overall, we found public sentiment toward vaccines and their manufacturers to be largely positive (58%), with a near equal split between negative (22%) and neutral (19%) sentiments. The sentiment trend was relatively steady over time and had minor variations, likely based on political and regulatory announcements and debates.

**Conclusions:**

The most frequently discussed COVID-19 AEFIs on social media were found to be broadly consistent with those reported in the literature and by government pharmacovigilance. We also detected potential safety signals from our analysis that have been detected elsewhere and are currently being investigated. As such, we believe our findings support the use of social media analysis to provide a complementary data source to conventional knowledge sources being used for pharmacovigilance purposes.

## Introduction

A number of vaccines for SARS-CoV-2 infection have been developed, found to be effective, and are now being rolled out at unprecedented speed and scale across the world. A major component of vaccine deployment strategies should be the use of robust surveillance systems to monitor for adverse effects following immunization (AEFIs) [1]. This is particularly important given the persisting concerns around vaccine hesitancy and that new vaccine technologies are being employed for the first time [2].

Postlicensure monitoring of AEFIs primarily consists of passive surveillance, whereby reports of AEFIs are collected and statistically analyzed by regulators (eg, Vaccine Adverse Event Reporting System in the United States and Yellow Card in the United Kingdom), and also in epidemiological studies [3]. However, there has recently been growing interest in exploring the use of social media data to supplement traditional pharmacovigilance methods [4]. These techniques could be particularly beneficial in low- and middle-income countries given their underdeveloped vaccine safety surveillance systems [5,6].

Recent studies have highlighted the potential of artificial intelligence–enabled social media analysis to complement conventional assessment methods, such as public surveys, and inform governments and institutions on public attitudes [7-9]. Social media analysis has yet to be used to explore commonly reported AEFIs with a vaccine against SARS-CoV-2 infection, which could help to identify potential safety signals not being identified elsewhere (eg, rarely reported side effects). Sentiment analysis can be useful to gauge public opinion around topics of interest, and peaks and valleys on sentiment trend graphs could inform deliberations on sensitivity analyses conducted prior to studies.

In this study, we aimed to assess the frequency and nature of COVID-19 AEFI-related mentions on social media and analyze public sentiment toward vaccines in the United Kingdom.

## Methods

### Data Sources

We used data from two of the most popular and representative social media platforms, namely, Facebook and Twitter. Facebook posts were obtained from CrowdTangle, and tweets were obtained from the COVID-19 Twitter Dataset (using a publicly available Twitter Application Processing Interface) [10,11]. We extracted English-language Facebook posts and tweets, posted in the United Kingdom from December 8, 2020 (the start of the United Kingdom’s COVID-19 vaccination campaign), to April 30, 2021, and thematically filtered these using a 2-step approach. The initial filter used predefined COVID-19–related keywords, and the resulting data set was used to assess COVID-19 AEFI-related mentions. The second filter used vaccine-related keywords, and this subset of data was used to analyze public sentiment toward vaccines and their manufacturers. A geographical filter for the United Kingdom was also applied across the data set (see Multimedia Appendix 1 for a detailed search strategy) [7].

### Vaccine AEFI Search Strategy

Our vaccine adverse effect search strategy was informed by AEFI reports received by the Yellow Card scheme and the Vaccine Adverse Event Reporting System, the national passive surveillance pharmacovigilance systems of the United Kingdom and the United States, respectively (see Multimedia Appendix 1 for a detailed search strategy). The frequency of grouped AEFI mentions was calculated and plotted. The output combined results from the Facebook and Twitter data sets (which had the “initial” filter applied) and represented them using horizontal stacked bar charts. The distribution of user posts was also obtained using descriptive statistics and density distribution plots.

### Hybrid Ensemble Model

**Figure 1:**
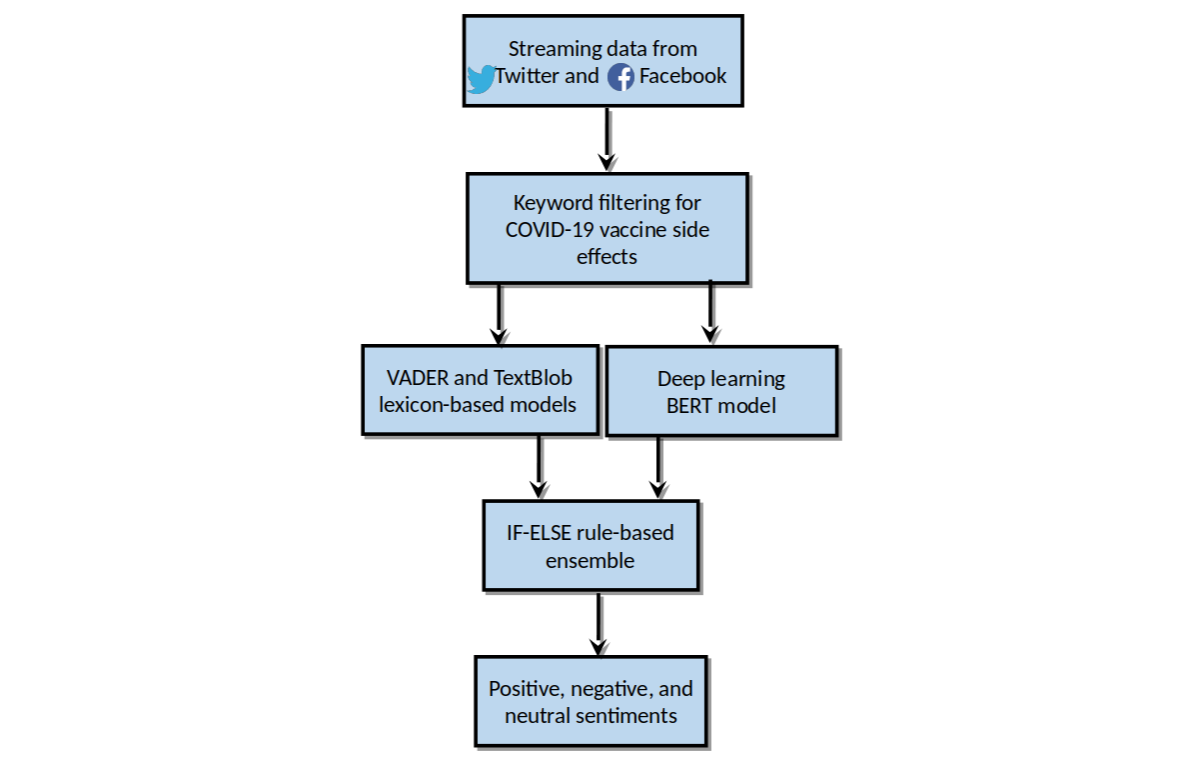
Hybrid ensemble sentiment analysis framework. BERT: Bidirectional Encoder Representations from Transformers; VADER: Valence Aware Dictionary for Sentiment Reasoning.

We also adapted and utilized our recently developed hybrid ensemble model (Figure 1), which combines state-of-the-art lexicon rule–based and deep learning–based approaches, to analyze sentiment trends relating to the main vaccines and their manufacturers since their rollout in the United Kingdom. Sentiment trend graphs were plotted, and average sentiment was calculated.

Our hybrid ensemble model utilized weighted averaging of the VADER (Valence Aware Dictionary for Sentiment Reasoning) and TextBlob lexicon-based models, resulting in the following weights: VADER × 0.45 + TextBlob × 0.55. A higher weight of 0.55 was assigned to TextBlob as it demonstrated marginally better accuracy compared to VADER for classifying positive sentiment. The averaged output was combined with the BERT (Bidirectional Encoder Representations from Transformers) deep learning model with the help of rule-based constructs. The lexicon models performed better for positive sentiments, and the BERT model provided better performance for neutral and negative sentiments. Therefore, they were combined through IF-ELSE rule-based programming constructs. If the output of lexicon-based weighted averaging was positive, then the IF-ELSE rules chose a positive output as the final output of the ensemble; otherwise, for neutral and negative sentiments, the output of the BERT model was preferred as the final ensemble output sentiment.

### Ethical Considerations

The data analyzed in this study were completely in the public domain, and no ethical review was necessary. A thorough assessment of the study’s privacy risk to individuals was conducted to ensure compliance with the General Data Protection Regulation. We also complied with best practices for user protection. Nonpublic material was not included in our data set. We did not share any posts or quotes from individuals, or names or locations of users that are not public organizations or entities.

## Results

Our COVID-19 AEFI search strategy identified 46,762 unique Facebook posts by 14,346 users and 74,644 tweets (excluding retweets) by 36,446 users over the 4-month period. This corresponded to an average of 3.26 (SD 6.40) posts per user on Facebook and 2.01 (SD 1.76) posts per user on Twitter. Density distribution plots showed the log-normal distributions of posts per user for both platforms (see Figures S1 and S2 in Multimedia Appendix 1).

**Figure 2:**
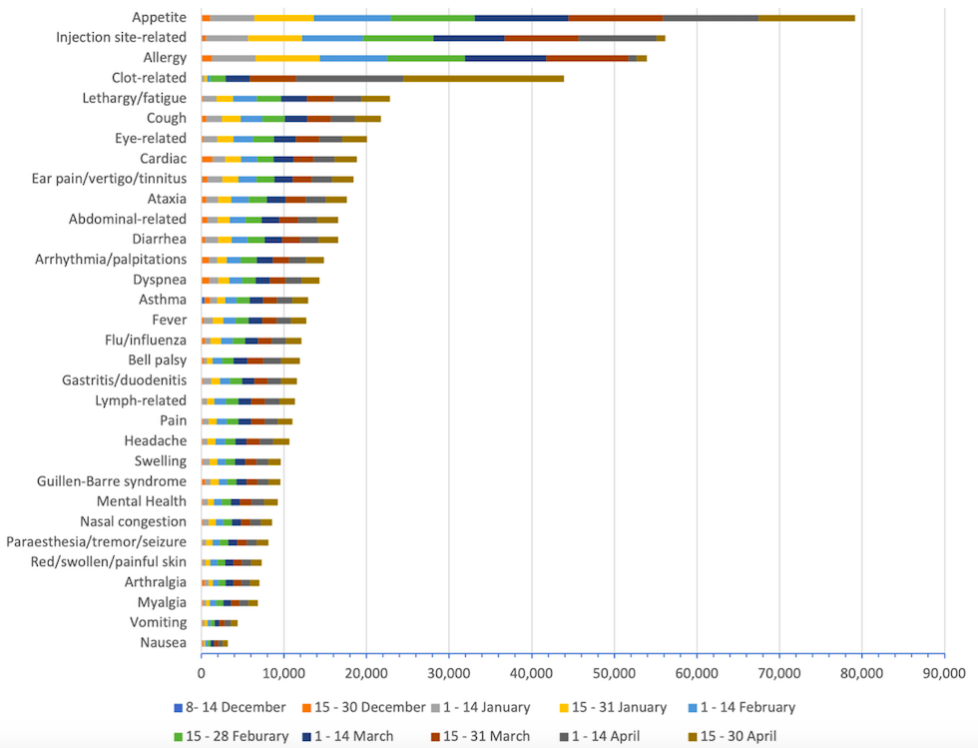
Stacked bar graph showing the number of mentions of each COVID-19 vaccine side effect over time on both Facebook and Twitter in the United Kingdom from December 2020 to April 2021.

Figure 2 shows the frequency of grouped AEFI mentions across Facebook and Twitter, divided into 2-week periods (see a detailed breakdown in Multimedia Appendix 1: Table S1, Figures S3 and S4). We identified an increasing number of mentions for each AEFI on social media over the period of study. The most frequent mentions were found to be symptoms related to appetite change (n=79,132, 14%), allergy (n=53,924, 9%), diarrhea (n=16,559, 3%), fever (n=12,707, 2%), headache (n=10,641, 2%), injection site (n=56,152, 10%), and clots (n=43,907, 8%) (see Table S1 in Multimedia Appendix 1). Less commonly mentioned AEFIs included Bell palsy (n=11,909, 2%) and Guillain-Barre syndrome (n=9576, 2%).

**Figure 3:**
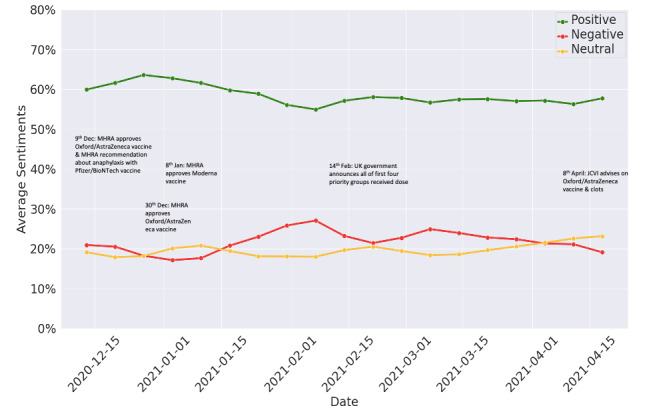
Average weekly public sentiments on COVID-19 vaccines on Facebook and Twitter in the United Kingdom from December 2020 to April 2021 with annotations of some key events. JCVI: Joint Committee on Vaccination and Immunisation; MHRA: Medicines and Healthcare Products Regulatory Agency.

Figure 3 shows the average weekly public sentiment on Twitter and Facebook. Overall, we found public sentiment toward vaccines to be largely positive (58%), with negative (22%) and neutral (19%) sentiment nearly equally split. The sentiment trend was relatively steady over time and had minor variations, likely based on political and regulatory announcements and debates.

## Discussion

### Principal Findings

We have identified and extracted a substantial number of social media posts relating to vaccines and possible AEFIs in the United Kingdom. Our analysis showed an increasing trend in the number of AEFI mentions over time and revealed that both established adverse events (eg, headaches and clots) and those still under investigation (eg, Bell palsy and Guillain-Barre syndrome) were being discussed.

The most frequently mentioned symptoms were broadly found to be similar to those most frequently reported in the Yellow Card system [12]. For example, the most commonly reported AEFI on Yellow Card was related to injection-site reactions and generalized symptoms (eg, fever, headache, lethargy, muscle ache, flu, vomit, nausea), which in our analysis accounted for 10% and 13% of the mentions, respectively. The number of clot-related AEFI mentions increased 2-fold from the end of March to mid-April, and approximately 2-fold again until the end of April, which correlates with the significant press coverage of reports in March 2021 on blood clots being associated with the Oxford-AstraZeneca vaccine [13]. In a recent study, the most common systemic side effects were found to be headache and fatigue, and the most common localized side effect was injection site–related pain, redness, or swelling [14]. The latter is consistent with our findings, where injection site pain or redness (n=56,152, 10%) was one of the most commonly mentioned AEFIs. It is interesting to note that we found more rarely reported AEFIs, such as Bell palsy, Guillain-Barre syndrome, and appetite changes, being discussed as frequently as more well-known side effects, such as headache, fever, and diarrhea. This can be useful for governments and institutions to detect potential safety signals and could enable further exploration of public perceptions toward rarer side effects and consideration of educational campaigns and interventions.

Public sentiment toward vaccines over the course of the vaccination rollout campaign has on the whole been consistently positive. This is in line with the successful uptake of vaccinations in the United Kingdom and important government and political announcements (examples can be found annotated in Figure 3). These findings indicate the potential for social media analysis to complement traditional surveys, both by informing their design and also corroborating findings [15].

Overall, this work has confirmed the opportunity for social media analysis to provide insights into public sentiments and complement more established pharmacovigilance efforts. It is important to note that we did not aim to identify new side effects; our objective was rather to monitor trends relating to currently reported ones. The trends identified can be useful for public health policy makers to identify which symptoms are being discussed most frequently. Further analysis can then be carried out on social media, alongside traditional surveys, to explore public perception relating to specific AEFIs.

### Strengths and Limitations

Our study is the first to assess trends in the number of AEFI mentions on both Facebook and Twitter. It employed our novel ensemble-based approach to analyze public sentiment toward vaccines over the course of the vaccination drive and has provided important insights into the number of posts relating to vaccines and AEFIs, trends in the number of AEFI mentions, and public sentiment toward vaccinations. Our AEFIs were informed by the Yellow Card system, and our keywords for filtering were informed by clinicians and a literature search. Our novel ensemble-based approach has been shown to robustly identify public sentiment over a period of time, for example, toward vaccinations and apps [8,9].

It is important to consider the limitations of our study to define challenges and inform future work. Social media users are by and large not representative of the wider UK population (eg, younger, wealthier, higher level of education); the age factor, in this case, is particularly significant given that the majority of COVID-19 vaccinations within the United Kingdom so far has been given to older age groups [8,9]. The selection bias from social media can be mitigated by using it as a complementary data source to conventional knowledge sources and by ensuring any search terms used to filter data sets are defined appropriately.

Another limitation is that it is difficult to ascertain which posts are specific to those who had received the vaccine (making them less useful if studying side-effect experiences) and would require deeper semantic and linked analysis with external trustworthy data sources, such as surveys and electronic health records. In addition, a proportion of social media users are known to be more vocal than others, which can skew study findings. Descriptive statistics and density plots of the distribution of user posts can therefore be useful to help contextualize findings (as was done in our study), while social network analysis could help identify clusters of users on a large scale.

Our study was also limited by its relatively small sample size, due to a stringent search strategy, restricting tweets to those with geotags and the use of the COVID-19 Twitter Dataset. We combined our results for Facebook and Twitter to mitigate this. For future work, we propose obtaining Academic Research access from Twitter and hydrating tweets at a large scale using an optimized search strategy. While this would be more time and labor intensive, it will provide more flexibility in the scope and breadth of tweets, as the data set would not be prefiltered for COVID-19. We also did not carry out any further manual labeling of social media posts to further train our ensemble-based model for extracting public sentiment due to resource limitations (however, it was trained in a previous study [8] to assess sentiment toward vaccines). Lastly, misinformation and fake news remain open challenges for social media analysis. Social media platforms have their own mechanisms to help tackle this issue; however, for future work, researchers can utilize techniques, such as social network analysis, to identify and remove clusters of users from their data sets.

### Conclusion

In summary, our work has shown it is possible to identify and interrogate large volumes of social media posts using our novel ensemble-based approach to generate insights into public sentiments toward vaccines and AEFIs. These can help develop a complementary data source to the conventional knowledge sources that are being used for pharmacovigilance purposes. In the future, governments and institutions should consider the opportunity to use social media analyses to aid pharmacovigilance efforts.

## Appendix

Multimedia Appendix 1 Detailed search strategy and results.

## Abbreviations

AEFI: adverse effect following immunization
BERT: Bidirectional Encoder Representations from Transformers
VADER: Valence Aware Dictionary for Sentiment Reasoning

## Data Availability

We have made the code and data used for data extraction and analysis openly available on GitHub [16].
