# Supplementary material for: Artificial Intelligence–Enabled Social Media Analysis for Pharmacovigilance of COVID-19 Vaccinations in the United Kingdom: Observational Study
Source: JMIR Public Health Surveill. 2022 May 27;8(5):e32543. doi: 10.2196/32543 (PMC9150729; doi:10.2196/32543)
Supplement: Multimedia Appendix 1 [file publichealth_v8i5e32543_app1.docx]

**Appendix A**

**Detailed Search Strategy:**

1^st^ Filter for Covid-19 related search terms:(Covid, COVD19, CoronavirusPandemic, COVID-19, 2019nCoV, CoronaOutbreak, coronavirus, WuhanVirus,covid19,coronaviruspandemic, covid-19, 2019ncov, coronaoutbreak, wuhanvirus)

2^nd^ Filter for vaccines and their manufacturer related search terms:

(immuniz, Vaccin, immunis, astrazeneca, Pfizer, moderna, BioNTech, Novavax, Sinopharm, Sinovac, Sanofi, sputnik, Oxford, Johnson & Johnson, Johnson and Johnson)

AEFI search terms:

1. Allergy OR allergic OR anaphylaxis OR Eczema OR itch OR rash [**Allergy**, combine with 18]
2. Arrhythmia OR palpitations OR tach (Combine with 8, under **Cardiac**-related)
3. Asthma,
4. Ataxia, [**Neurological**, combine with 5, 15, 16, 27]
5. Bell’s palsy
6. Breathless OR breath OR sinus OR nasal OR dyspnoea OR throat OR mouth
7. Cough
8. cardiac OR heart OR cardio OR chest
9. Ear pain OR vertigo OR tinnitus OR dizz OR balance
10. Diarrhea OR loose stool (**Gastrointestinal**, with 11, 12, 13
11. Abdo
12. Gastritis OR duodenitis OR reflux OR gord
13. Appetite
14. Fever OR chills OR chilly OR pyrexia OR sweat OR temp OR flush OR febrile [**Systemic**, combine with 21, 22]
15. GBS OR Guillain-Barre,
16. Headache OR head pain OR migraine
17. (Injection OR site) AND (pain OR red OR swell OR bruis OR urticaria) [Injection Site]
18. Skin AND (pain OR red OR swell OR bruis OR urticaria)
19. Pain,
20. Sleep OR dream OR anxiety OR anxious OR panic OR psych OR mood OR depress OR insomnia OR mental OR sad OR unhappy
21. Lethargy OR malaise OR fatigue OR tired,
22. Muscle AND (ache OR pain) OR myalgia [Combine with 31]
23. Lymph,
24. Nasal congestion OR block nose,
25. Swelling
26. Eye AND (pain OR red OR swell OR allergy OR itch OR irrit), Optic neuritis OR photophobia OR blind OR blur
27. Paresthesia OR tingl OR tremor OR seizure
28. Flu OR influenza
29. Vomit (combine with 30)
30. Nausea
31. Joint pain OR arthralgia
32. stroke, ITP, thrombocytopenia, clot, low platelets, thrombosis, CVST, DVT, PE, haemorrhage, embolism, infarct

**Figure S1: Distribution of Facebook posts per user**


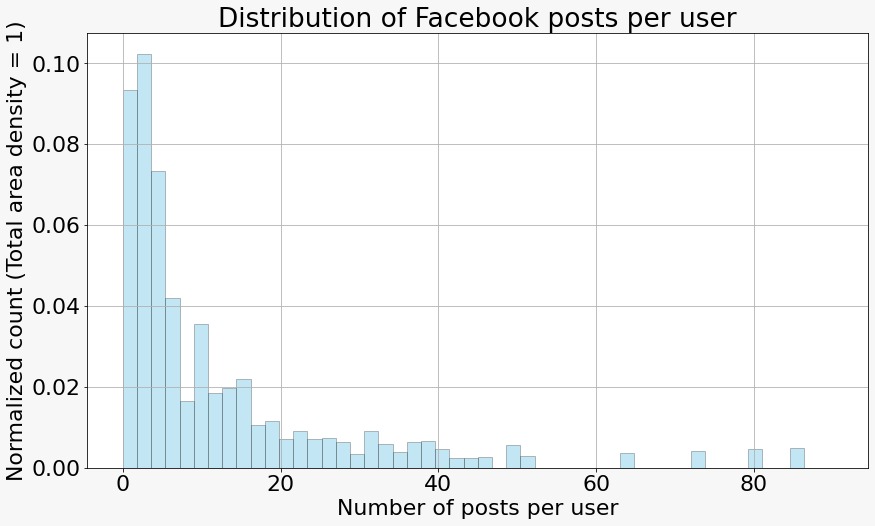


**Figure S2: Distribution of Twitter tweets per user**


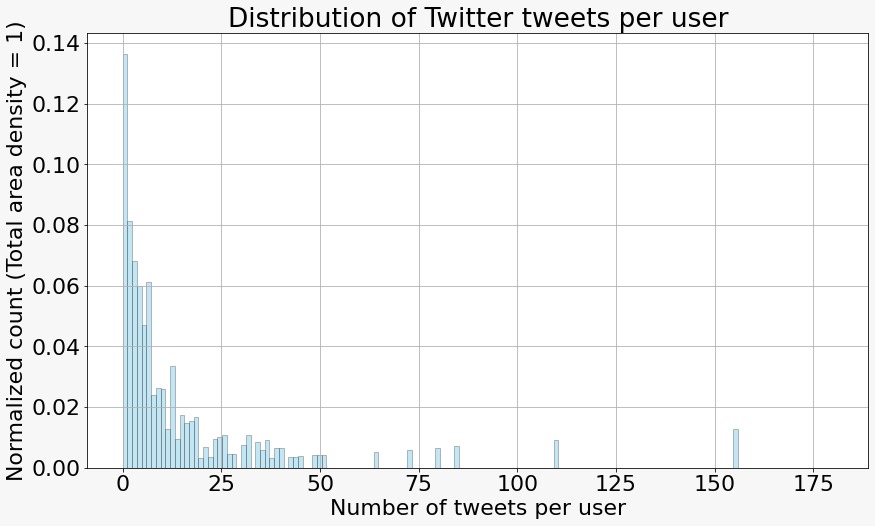


**Figure S3: Stacked bar graph showing number of mentions of each COVID-19 vaccine side effect over time, on both Facebook and Twitter, in the UK, from December 2020 to April 2021 (top 16)**


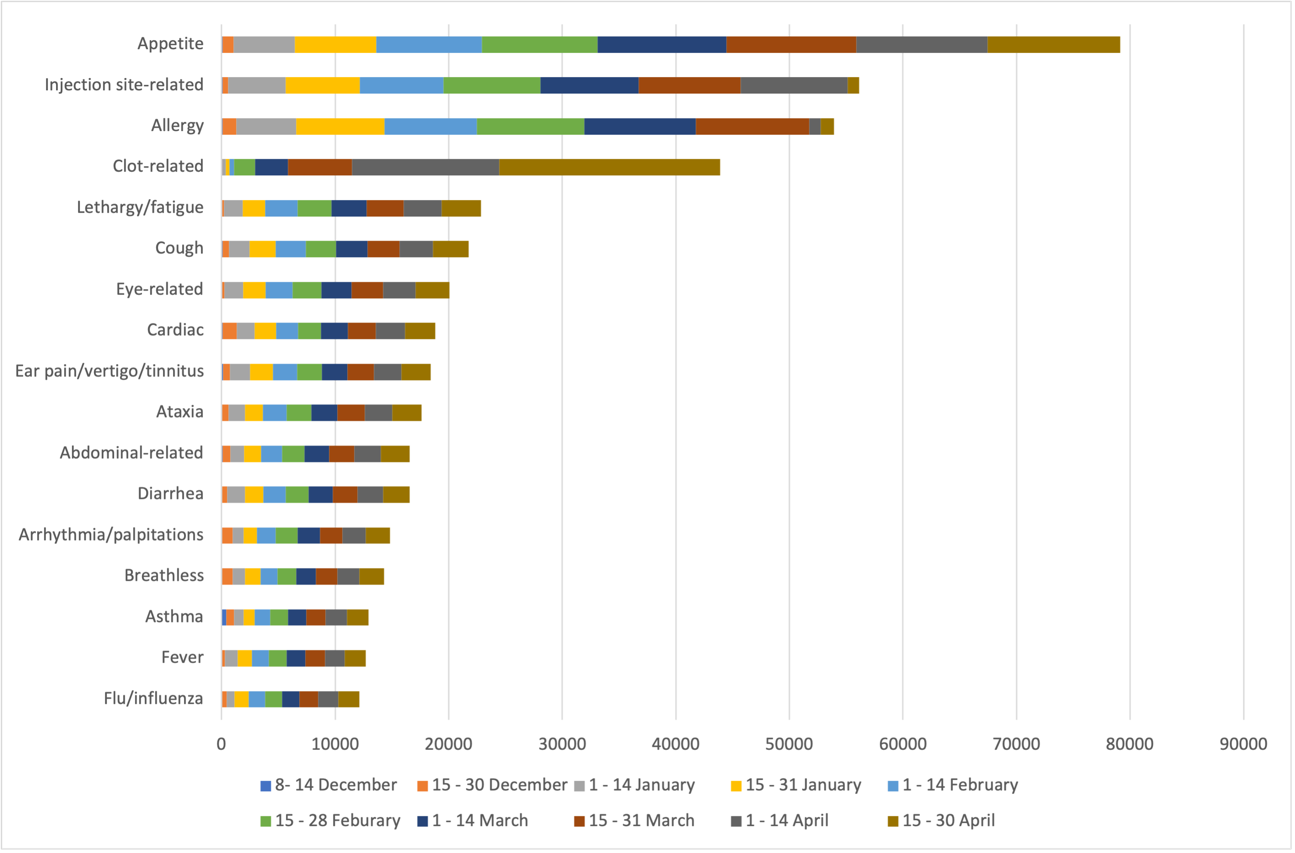


**Figure S4: Stacked bar graph showing number of mentions of each COVID-19 vaccine side effect over time, on both Facebook and Twitter, in the UK, from December 2020 to April 2021 (bottom 16)**


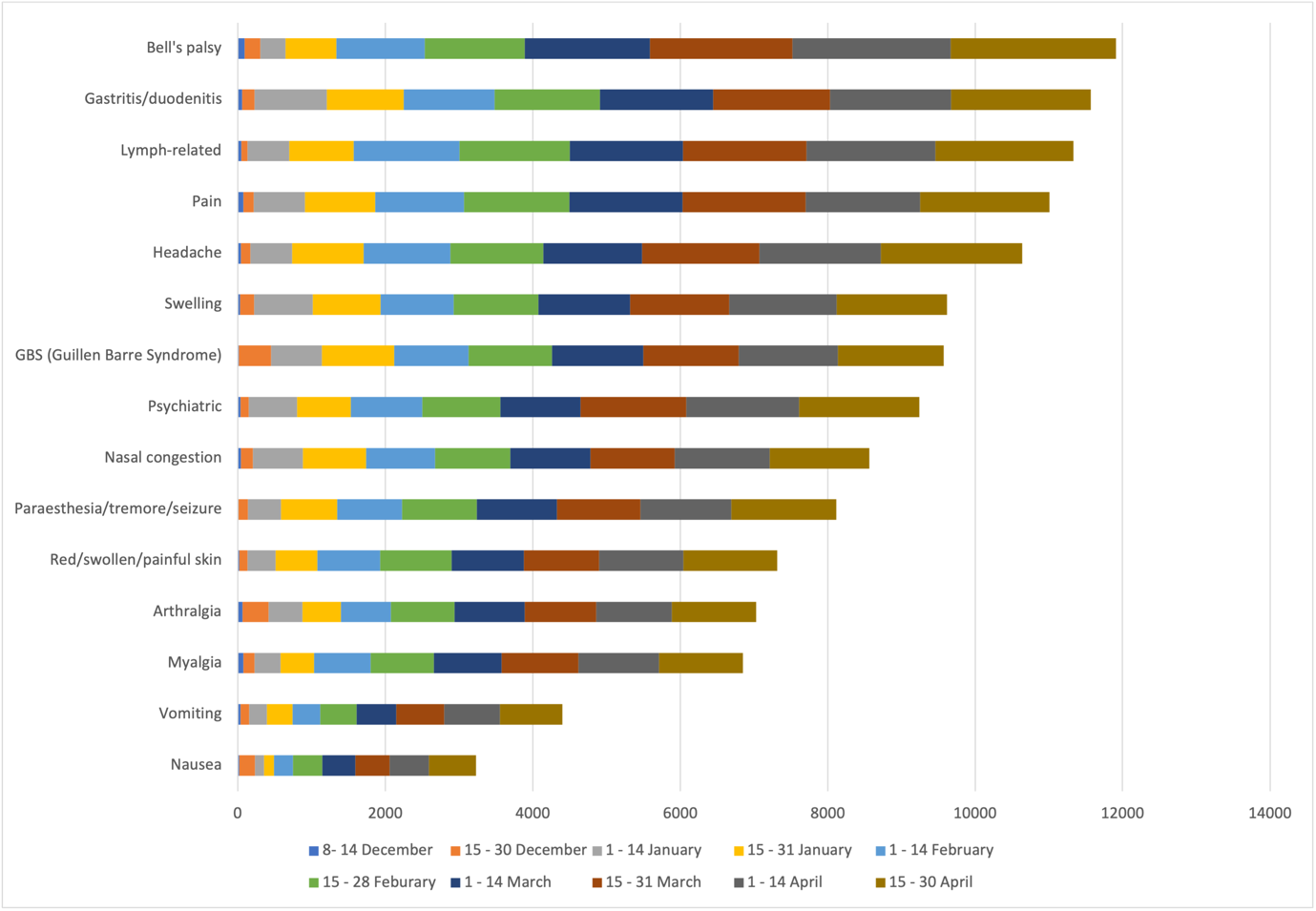


**Table S1: AEFI grouped, and total number of mentions and percentages presented**

| **AEFI** | **Total number of mentions** | **Percentage of mentions** |
| --- | --- | --- |
| Allergy OR allergic OR anaphylaxis OR Eczema OR  itch OR rash | 53924 | 9% |
| Arrhythmia OR palpitations OR tach | 14821 | 3% |
| Asthma | 12936 | 2% |
| Ataxia | 17606 | 3% |
| Bell’s palsy | 11909 | 2% |
| Breathless OR breath OR sinus OR nasal OR  dyspnoea OR throat OR mouth | 14287 | 2% |
| Cough | 21752 | 4% |
| cardiac OR heart OR cardio OR chest | 18819 | 3% |
| Ear pain OR vertigo OR tinnitus OR dizz OR  balance | 18421 | 3% |
| Diarrhea OR loose stool | 16559 | 3% |
| Abdo | 16574 | 3% |
| Gastritis OR duodenitis OR reflux OR gord | 11569 | 2% |
| Appetite | 79132 | 14% |
| Fever OR chills OR chilly OR pyrexia OR sweat OR  temp OR flush OR febrile | 12707 | 2% |
| GBS OR Guillain-Barre, | 9576 | 2% |
| Headache OR head pain OR migraine | 10641 | 2% |
| (Injection OR site) AND (pain OR red OR swell OR  bruis OR urticaria) | 56152 | 10% |
| Skin AND (pain OR red OR swell OR bruis OR  urticaria) | 7314 | 1% |
| Pain, | 11006 | 2% |
| Sleep OR dream OR anxiety OR anxious OR panic OR  psych OR mood OR depress OR insomnia OR mental | 9241 | 2% |
| Lethargy OR malaise OR fatigue OR tired, | 22829 | 4% |
| Muscle AND (ache OR pain) OR myalgia | 6849 | 1% |
| Lymph, | 11331 | 2% |
| Nasal congestion OR block nose, | 8567 | 1% |
| Swelling | 9617 | 2% |
| Eye AND (pain OR red OR swell OR allergy OR itch  OR irrit), Optic neuritis OR photophobia OR blind OR blur | 20070 | 3% |
| Paresthesia OR tingl OR tremor OR seizure | 8115 | 1% |
| Flu OR influenza | 12109 | 2% |
| Vomit, | 4401 | 1% |
| Nausea, | 3232 | 1% |
| Joint pain OR arthralgia | 7028 | 1% |
| stroke, ITP, thrombocytopenia, clot, low  platelet, thrombosis, CVST, DVT, PE, haemorrhage, embolism, infarct | 43907 | 8% |

**Table S2: AEFIs grouped, with number of mentions for each 2-week period**

|  | First two Dec | Sec Two Week Dec | First two Jan | Second two week Jan | First two week Feb | Sec two week Feb | First two week March | Second two week March | First two week April | Second two week April |
| --- | --- | --- | --- | --- | --- | --- | --- | --- | --- | --- |
| Allergy OR allergic OR anaphylaxis OR Eczema OR itch OR rash | 89 | 1201 | 5265 | 7797 | 8112 | 9461 | 9842 | 9959 | 1012 | 1186 |
| Arrhythmia OR palpitations OR tach | 59 | 895 | 985 | 1150 | 1650 | 1953 | 1961 | 1975 | 2050 | 2143 |
| Asthma | 401 | 675 | 845 | 986 | 1350 | 1594 | 1624 | 1684 | 1864 | 1913 |
| Ataxia | 43 | 568 | 1430 | 1578 | 2109 | 2189 | 2286 | 2389 | 2451 | 2563 |
| Bell’s palsy | 91 | 215 | 341 | 689 | 1201 | 1354 | 1697 | 1937 | 2143 | 2241 |
| Breathless OR breath OR sinus OR nasal OR dyspnoea OR throat OR mouth | 45 | 935 | 1091 | 1341 | 1506 | 1642 | 1753 | 1874 | 1957 | 2143 |
| Cough | 78 | 563 | 1840 | 2258 | 2670 | 2684 | 2751 | 2843 | 2941 | 3124 |
| cardiac OR heart OR cardio OR chest | 87 | 1257 | 1567 | 1871 | 1961 | 2021 | 2376 | 2442 | 2564 | 2673 |
| Ear pain OR vertigo OR tinnitus OR dizz OR balance | 121 | 631 | 1764 | 1989 | 2150 | 2167 | 2238 | 2341 | 2449 | 2571 |
| Diarrhea OR loose stool | 39 | 463 | 1540 | 1641 | 1942 | 2049 | 2124 | 2156 | 2264 | 2341 |
| Abdo | 97 | 657 | 1231 | 1465 | 1890 | 1951 | 2163 | 2234 | 2345 | 2541 |
| Gastritis OR duodenitis OR reflux OR gord | 56 | 169 | 981 | 1046 | 1230 | 1432 | 1534 | 1586 | 1641 | 1894 |
| Appetite | 86 | 968 | 5404 | 7143 | 9302 | 10214 | 11326 | 11453 | 11563 | 11673 |
| Fever OR chills OR chilly OR pyrexia OR sweat OR temp OR flush OR febrile | 49 | 259 | 1104 | 1258 | 1497 | 1563 | 1643 | 1721 | 1759 | 1854 |
| GBS OR Guillain-Barre, | 12 | 438 | 689 | 981 | 1012 | 1127 | 1243 | 1291 | 1346 | 1437 |
| Headache OR head pain OR migraine | 39 | 129 | 571 | 967 | 1176 | 1259 | 1342 | 1594 | 1643 | 1921 |
| (Injection OR site) AND (pain OR red OR swell OR bruis OR urticaria) | 102 | 459 | 5101 | 6523 | 7365 | 8532 | 8647 | 8963 | 9453 | 1007 |
| Skin AND (pain OR red OR swell OR bruis OR urticaria) | 19 | 114 | 378 | 569 | 851 | 967 | 983 | 1021 | 1143 | 1269 |
| Pain, | 75 | 143 | 694 | 953 | 1203 | 1431 | 1532 | 1673 | 1549 | 1753 |
| Sleep OR dream OR anxiety OR anxious OR panic OR psych OR mood OR depress OR insomnia OR mental | 38 | 112 | 654 | 731 | 967 | 1059 | 1087 | 1435 | 1531 | 1627 |
| Lethargy OR malaise OR fatigue OR tired, | 29 | 169 | 1656 | 1987 | 2870 | 2961 | 3102 | 3246 | 3356 | 3453 |
| Muscle AND (ache OR pain) OR myalgia | 76 | 149 | 352 | 458 | 769 | 853 | 921 | 1043 | 1094 | 1134 |
| Lymph, | 49 | 83 | 564 | 873 | 1436 | 1496 | 1536 | 1674 | 1753 | 1867 |
| Nasal congestion OR block nose, | 39 | 163 | 682 | 857 | 934 | 1021 | 1086 | 1143 | 1291 | 1351 |
| Swelling | 29 | 194 | 791 | 923 | 989 | 1149 | 1243 | 1348 | 1457 | 1494 |
| Eye AND (pain OR red OR swell OR allergy OR itch OR irrit), Optic neuritis OR photophobia OR blind OR blur | 49 | 216 | 1652 | 1961 | 2359 | 2548 | 2657 | 2784 | 2856 | 2988 |
| Paresthesia OR tingl OR tremor OR seizure | 8 | 128 | 452 | 759 | 879 | 1014 | 1089 | 1134 | 1231 | 1421 |
| Flu OR influenza | 29 | 421 | 689 | 1249 | 1432 | 1499 | 1543 | 1641 | 1763 | 1843 |
| Vomit, | 38 | 115 | 239 | 351 | 375 | 493 | 539 | 647 | 761 | 843 |
| Nausea, | 18 | 215 | 121 | 137 | 259 | 394 | 451 | 463 | 531 | 643 |
| Joint pain OR arthralgia | 64 | 351 | 463 | 521 | 676 | 863 | 953 | 971 | 1023 | 1143 |
| stroke, ITP, thrombocytopenia, clot, low platelet, thrombosis, CVST, DVT, PE, haemorrhage, embolism, infarct | 12 | 21 | 321 | 350 | 391 | 1861 | 2891 | 5634 | 12963 | 19463 |
